# Post-Intensive Care Syndrome Awareness and Communication: Surveys of ICU Providers and Patients

**DOI:** 10.1016/j.chest.2025.08.048

**Published:** 2025-10-10

**Authors:** Mark L. Rolfsen, Matthew F. Mart, Hannah Kieffer, David Krasinski, Timothy D. Girard, Lauren E. Ferrante, Robert L. Owens, Ana Lucia Fuentes, Nathan Brummel, Carla M. Sevin, John P. Kress, Jaspal Singh, Sukhvinder Nagi, Kevin Shaw, Edward Qian, James C. Jackson, Christopher G. Hughes, Pratik Pandharipande, Mayur Patel, Tom Elasy, E. Wesley Ely

**Affiliations:** a Critical Illness, Brain Dysfunction, and Survivorship (CIBS) Center, Nashville TN; b Division of Allergy, Pulmonary and Critical Care Medicine, Department of Medicine, Vanderbilt University Medical Center, Nashville TN; c Division of General Internal Medicine, Department of Medicine, Vanderbilt University Medical Center, Nashville TN; d Department of Medicine, Vanderbilt University Medical Center, Nashville TN; e Department of Anesthesiology, Vanderbilt University Medical Center, Nashville TN; f Section of Surgical Sciences, Division of Acute Care Surgery, Department of Surgery, Vanderbilt University Medical Center, Nashville TN; g Geriatric Research, Education, and Clinical Center, Veterans Affairs Tennessee Valley Health System, Nashville TN; h Center for Research, Investigation, and Systems Modeling of Acute Illness, Department of Critical Care Medicine, University of Pittsburgh School of Medicine, Pittsburgh, PA; i Division of Pulmonary, Critical Care and Sleep Medicine, Department of Medicine, Yale University School of Medicine, New Haven, CT; j Division of Pulmonary, Critical Care and Sleep Medicine, Department of Medicine, University of California San Diego, La Jolla, CA; k Department of Anesthesia and Critical Care Medicine, Kaiser Permanente Medical Group, Northern California, San Jose CA; l Division of Critical Care Medicine, Department of Medicine, Scripps Memorial Hospital Encinitas, Encinitas, CA; m Division of Pulmonary, Critical Care, and Sleep Medicine, The Ohio State University College of Medicine, Columbus, OH; n Division of Pulmonary and Critical Care, Department of Medicine, University of Chicago, Chicago, IL; o Atrium Health and Levine Cancer Institute, Carolinas Medical Center and Atrium Health, Charlotte, NC

**Keywords:** acute respiratory failure, Alzheimer’s disease-related dementias, delirium, disabilities, ICU communication, neurocognitive impairment, post-intensive care syndrome

## Abstract

**Background:**

Survivors of critical illness often experience new or worsening impairments in various domains of health after discharge, collectively referred to as post-intensive care syndrome (PICS). Although this condition is common, it remains unclear whether providers are communicating routinely about survivorship and PICS to patients and families and whether patients are remembering these conversations.

**Research Question:**

How often do ICU providers discuss the concept of PICS with at-risk patients or families, and how often do patients remember being told about the concept of PICS?

**Study Design and Methods:**

We distributed online surveys to ICU health care providers at 9 US institutions and to patients who survived critical illness in the preceding year at a single site.

**Results:**

We collected a convenience sample of 382 provider responses and 148 patient responses. The providers were registered nurses (53.7%), physician fellows or attending physicians (33%), and advanced practice providers (13.4%). Patients predominantly had been admitted to surgical (41.1%), cardiovascular (41.1%), and medical (14.4%) ICUs. We found that 73.8% of providers reported having previously heard the term *post-intensive care syndrome*. In comparison, only 16.6% of patients remembered ever being told the term. When asked how often they would discuss with patients or families the possibility of any new or worsening impairments after critical illness, less than one-third of providers (29.9%) said they do so at least one-half of the time. Only about one-third of patients (35.6%) remembered such conversations.

**Interpretation:**

Our results show that awareness of PICS is inconsistent among providers and low among patients. Few ICU team members reported routinely talking to patients or families about the common, disabling impairments that often occur after critical illness. Few patients remembered being told about the possibility of PICS. Further investigation is needed to determine how best to improve this communication gap.

## Graphical Abstract

**Figure:**
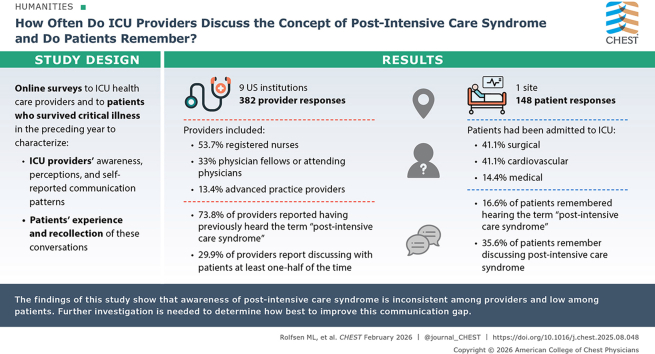

**Take-Home Points**

**Research Question:** How often do providers discuss the concept of post-intensive care syndrome (PICS) with patients or families and how often do patients remember being told about the concept of PICS?

**Results:** Less than one-third of providers in our geographically diverse sample reported discussing the concept of PICS with at-risk patients or families at least one-half of the time, and only about one-third of patients in this single-site sample remembered being told about these possible health problems that can follow critical illness.

**Interpretation:** Our results show that communication about PICS is insufficient for the needs of patients and could result from gaps in provider communication practices or from a patient’s ability to remember and comprehend these conversations.

Communication is considered an integral part of high-quality medical care. Outside of the ICU, quality communication long has been associated with wide benefits including patient satisfaction, understanding, and adherence.^1^, ^2^, ^3^, ^4^ Inversely, poor medical communication and lower scores on communication skills testing have been associated with worse patient satisfaction and higher likelihood of malpractice claims.^5^^,^^6^ Many studies have focused on communication during goals-of-care and end-of-life conversations for patients with serious illness such as terminal cancer or end-stage organ dysfunction.^7^ These investigations have demonstrated that sometimes as part of palliative care interventions, engaging in conversations that improve a patient’s prognostic understanding can reduce goal-discordant care near the end of life and can improve quality of life.^8^^,^^9^

Within the ICU, studies have demonstrated the importance, and also the difficulty, of successfully communicating survival and short-term prognosis to patients and families.^10^, ^11^, ^12^, ^13^ Yet, it has become clear that patient priorities often are focused not only on survival, but on maintaining adequate function and a self-perceived quality of life after discharge from the ICU.^14^^,^^15^ Societal recommendations on shared decision-making highlight this by including discussion of long-term functional impairments as a key communication skill.^16^ These long-term functional impairments after critical illness, including new or worsening health problems in cognitive, physical, and mental health domains, are referred to collectively as post-intensive care syndrome (PICS).^17^ PICS can occur in up to one-half of survivors of the ICU and has received increased research attention over the past 25 years,^18^, ^19^, ^20^ but it is unclear if the possibility of these impairments is communicated to survivors.

Interviews with intensivists have shown that discussions of long-term functional outcomes, that is, PICS, are considered by many to be a professional responsibility, yet several barriers have been identified.^21^ These include prognostic uncertainty, unrealistic optimism by surrogates, and lack of experience in communicating this topic.^21^ Currently few data are available regarding awareness of PICS^22^ or how often conversations about survivorship after critical illness occur.^23^, ^24^, ^25^ We sought to better characterize the communication of PICS by collecting survey data on ICU providers’ awareness, perceptions, and self-reported communication patterns and on patients’ experience and recollection of such conversations.

## Study Design and Methods

### Survey Design

**Figure 1:**
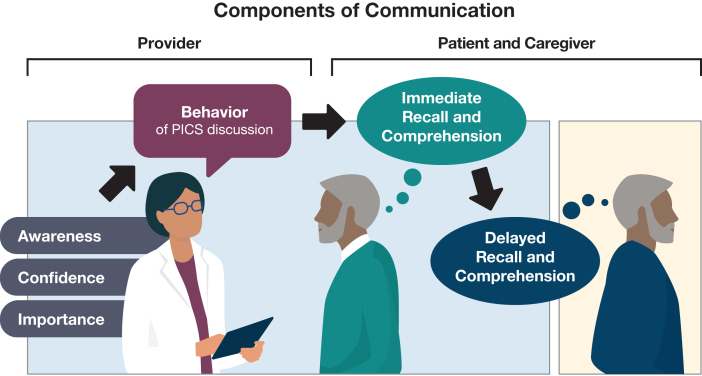
Nonexhaustive map showing components of communication. The health care provider must be aware of the content, feel confident in discussing the content, and perceive it as important. Their behavior while discussing the content must result in immediate recall and comprehension by the patient or caregiver. To be most effective, patients must recall and comprehend this information later. This figure does not include all complexities of successful communication, but rather gives a framework for our surveys. PICS = post-intensive care syndrome.

We performed a survey-based study of ICU providers and patients who recently survived critical illness. A survey for each of these 2 groups was created after several rounds of feedback from researchers and clinicians. Feedback focused on content validity, language and terminology, and adequacy of responses. Surveys were designed to be completed in < 2 minutes for providers and < 5 minutes for patients. Full surveys are available in e-Appendix 1. These surveys were rooted in a conceptual framework of communication in which ICU providers first must have awareness of the content, confidence in communicating the content, and perceived importance in communicating the content. After the information is delivered (ie, the behavior of discussing PICS), the patient or surrogate must comprehend and remember the content (Fig 1).

Provider surveys included 11 multiple choice questions. Two questions were about respondent characteristics. The remainder intended to capture components of the aforementioned pathway: awareness of the content (2 questions), confidence in being able to communicate the content (1 question), perceived importance of communicating the content (1 question), and behavior or practice patterns in communicating the content (5 questions).

Patient surveys included 14 multiple choice questions and 1 open-ended question. Three questions were about respondent characteristics. One question regarded whether respondents remembered being told the term *post-intensive care syndrome*. Four questions were about remembering discussions of new or worsening health problems after critical illness, including 1 about general problems and 1 for each of the 3 standard domains of PICS (physical, cognitive, and mental health) using basic language. Five questions were about the respondents’ current health status and struggles after critical illness. One question was about their perception of whether more conversations about recovery should occur in the ICU.

### Target Populations and Distribution

To survey providers, we asked ICU leaders at 9 institutions to distribute emails targeted at registered nurses (RNs), advanced practice providers (APPs), critical care fellows, and attending physicians. Emails contained links to an anonymous online survey. Providers were informed that these surveys were for research and that their response implied consent to use their answers. Given the nature of these email distribution lists, the number of recipients was unable to be tracked.

For patients, we identified recent adult survivors of critical illness, a cohort previously described to be at high risk of PICS.^26^, ^27^, ^28^ Patients were identified using electronic health record reports. We included in the report adult patients (age 18 years or older) admitted to an ICU at Vanderbilt University Medical Center (Nashville, Tennessee) in the preceding 12 months, not documented to be deceased, who had experienced respiratory failure or shock and elected to receive research invitations. Respiratory failure was defined as documented flowsheet value for invasive mechanical ventilation. Shock was identified as a documented flowsheet value for common vasopressors at Vanderbilt University Medical Center (norepinephrine, epinephrine, or vasopressin). Patients with documented history of dementia and those whose primary language was not English were excluded.

We sent recruitment invitations to patients identified by this report via electronic health record. If the patient was interested, an email was sent with a unique link to an online survey. We obtained consent electronically before the survey, sent up to 3 email reminders on a weekly basis or until the survey was completed, and offered a gift card at the end of the survey. Both provider and patient surveys were performed on a secure, web-based software platform (Research Electronic Data Capture).^29^

### Conduct and Reporting

The Vanderbilt University Medical Center institutional review board approved both survey studies (Identifier: 240117 for providers under exempt status; and Identifier: 240427 for patients). Design and reporting use Enhancing the Quality and Transparency of Health Research Network guidelines on Standards for Reporting Qualitative Research studies.^30^

### Statistical Analysis

For both groups, we report descriptive statistics using medians with interquartile ranges for continuous variables and frequencies with proportions for categorical variables where appropriate. For providers, we included all RNs, APPs, fellows, and attending physicians who completed at least 1 question. For patients, we included all patients who had at least 1 ICU admission during the 12 months preceding the invitation. We analyzed completed questions and reported missingness. The χ^2^ goodness-of-fit test was performed for prespecified categorical variables. We used Microsoft Excel software and R version 4.4.2 software (R Foundation for Statistical Computing).^31^

## Results

### ICU Provider Characteristics

We distributed provider surveys from February 1, 2024, through March 5, 2025. Institutions were academic (n = 6), mixed academic and nonacademic (n = 2), and nonacademic (n = 1). These were located throughout most US regions, including the West (n = 3), Southeast (n = 2), Midwest (n = 2), and Northeast (n = 2).

We obtained 382 survey responses that met inclusion criteria. No response rate was able to be calculated because of the nature of the distribution lists. There was a median of 12 respondents per institution (interquartile range, 5.5-90.5 respondents). This included 205 RNs, 90 attending physicians, 51 APPs, and 36 fellows. One-half of respondents (53.8%) worked primarily in a medical ICU (Table 1). Less than 1% of responses were missing.

**Table 1:** Characteristics of ICU Providers (N = 382)

Characteristic	Data
No. of institutions	9
Respondents per institution	12 (5.5-90.5)
Role of respondents	
RN	205 (53.7)
Attendings	90 (23.6)
APP	51 (13.4)
Fellow	36 (9.4)
Primary ICU of respondents	
Medical	205 (53.8)
Cardiovascular or cardiac	63 (16.5)
Surgical	21 (5.5)
Trauma	15 (3.9)
Other	27 (7.1)
Combination of ≥ 2	50 (13.1)

Data are presented as No. (%) or median (interquartile range) unless otherwise indicated. APP = advanced practice provider; RN = registered nurse.

### Patient Characteristics

We distributed patient surveys from October 1, 2024, through March 5, 2025. Electronic health record invitations were sent to 1,600 patients identified by our report. Messages were viewed by 523 patients; 285 responded (228 patients were interested and 57 patients were not interested). We obtained consent from 148 patients, with 147 patients completing a survey. Therefore, completion rate for initial invitations was 9.2%. One patient had never been admitted to an ICU. Among the 146 patients included in our analysis, median age was 56 years, 47.9% were female, and 93.8% had a documented race of White or Caucasian. Most recently had been admitted to a surgical ICU (41.1%), cardiovascular ICU (41.1%), or medical ICU (14.4%).

Baseline and clinical characteristics are shown in Table 2. We manually adjudicated 81.5% of patients as having respiratory failure or shock during the most recent ICU admission. The median maximum Sequential Organ Failure Assessment score^32^ during the ICU admission was 7, median total ICU length of stay was 4 days, and 37% experienced at least 1 episode of delirium as documented by the Confusion Assessment Method for the ICU.^33^

**Table 2:** Characteristics of Patients Treated in the ICU (N = 146)

Variable	Data
Age, y	56 (42-65)
Female sex	70 (47.9)
Race or ethnicity in chart	
White	137 (93.8)
Black	7 (4.8)
Hispanic or Latino	2 (1.4)
Most recent ICU type	
Cardiovascular ICU	60 (41.1)
Surgical ICU	60 (41.1)
Medical ICU	21 (14.4)
Cardiac	4 (2.7)
Trauma ICU	1 (0.7)
> 1 admission in past year	26 (17.8)
Respiratory failure, shock, or both	119 (81.5)
Maximum SOFA score during most recent ICU stay	7 (5-10)
Duration of ICU stay, d	4 (2-6)
Formal palliative care consultation during ICU stay or discharge to palliative care unit	2 (1.4)
Code status	
Full code	100 (68.5)
DNR	3 (2.1)
None listed	43 (29.5)
Positive results of delirium assessment during ICU stay	54 (37)
Positive reuslts of CAM-ICU on day of ICU discharge	1 (0.7)
Duration of delirium by CAM-ICU, d	0 (0-1)
Coenrollment in delirium- or PICS-related studies	12 (8.2)

Data are presented as No. (%) or median (interquartile range). CAM-ICU = Confusion Assessment Method for the ICU; DNR = do not resuscitate; PICS = post-intensive care syndrome; SOFA = Sequential Organ Failure Assessment.

### Awareness, Confidence, and Importance

In our analysis of the responses from RNs, APPs, fellows, and attending physicians, 73.8% of providers had heard of the term *post-intensive care syndrome* before the survey. This awareness ranged from 56% in RNs to 86% in APPs to 97.6% in fellows and attending physicians (*P* < .00001). In contrast, only 16.6% of patients remembered being told the term *post-intensive care syndrome*. Of providers, 31.7% reported feeling good or excellent when asked about their confidence in communicating to patients and families the likeliness of having new or worsening impairments after critical illness. This included 29.9% of RNs, 28% of APPs, and 36% of fellows and attending physicians (*P* = .43). When asked about whether they believed their ICU considered it important to discuss the concept of PICS with patients and families before ICU discharge, 53.1% either agreed or strongly agreed. This included 47.8% of RNs, 60.8% of APPs, and 58.7% of fellows and attending physicians (*P* = .078). Among respondents, 46.3% indicated that no one currently held responsibility for holding these conversations. The second most common answer was attending physicians (18.1%).

### PICS Communication

Providers were given a clinical vignette about a patient who required mechanical ventilation and vasopressors with several days of delirium and who was expected to return home and potentially to full employment. When asked how often they would counsel this patient or family about any new or worsening impairments that might occur after critical illness, 29.9% said they would do so more than one-half of the time. When asked about how often they would do so for specific domains of PICS, 24.6% reported doing so more than one-half of the time for cognitive problems, 55.4% for physical problems, and 22.8% for mental health problems.

**Figure 2:**
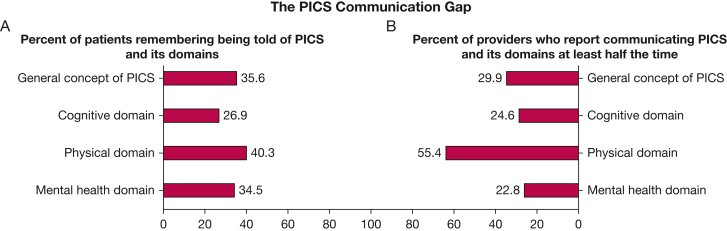
A, Bar graph showing the percent of patients who remember being told that they might have any new or worsening impairments after critical illness (“General concept of PICS”) or specific domains of new or worsening impairments. B, Graph showing the percent of providers who reported communicating to patients or families at least one-half of the time about the potential for any new or worsening impairments after critical illness (“General concept of PICS”) or specific domains of new or worsening impairments. The gap in the middle represents the percentage of patients who do not remember these conversations plus the percentage of providers who report communicating these ideas to patients or families less than one-half of the time. PICS = post-intensive care syndrome.

Patients were asked if they remember being told about post-ICU impairments at any point during the most recent hospitalization that included an ICU admission. When asked if they remember being told that they might not return to the same baseline function (including thinking, physical abilities, or mental health) after the ICU admission as they were before, 35.6% responded “yes.” When asked if they remember being told that they might develop new or worsening impairments in various domains of health, with basic language examples, 26.9% said “yes” to cognitive health problems, 40.3% said “yes” to physical health problems, and 34.5% said “yes” to mental health problems (Fig 2).

### Patient Experiences After Critical Illness

When asked if they were surprised at how challenging the recovery from critical illness has been, 45.2% of patients said “yes.” When asked if they were close to functioning at the same level as before ICU admission, 70.6% said “yes.” When asked about specific domains of impairment, 33.6% reported having struggled with cognitive problems, 37.9% reported having struggled with physical problems, and 32.9% reported having struggled with mental health problems. Overall, 56.2% of patients reported struggling with at least 1 domain of PICS since the critical illness. When asked whether they wish there were more conversations about recovery from critical illness before leaving the ICU, 58.2% reported agreeing or strongly agreeing.

## Discussion

In this survey-based study to assess awareness, perceptions, and communication of PICS among ICU providers and patients, we found that overall awareness was good among self-selected physicians and APPs, but poor among nurses and patients. Providers had low confidence in their ability to communicate the likeliness of PICS; fewer than 1 in 3 providers reported communicating PICS to patients or families at least one-half of the time even when presented with a vignette of a high-risk patient expecting to return home and to employment. Patients seldom remember being counseled about impairments after ICU discharge, yet wish they were told more during the ICU stay. Surprisingly, only one-half of providers report that their ICU considers it important to have conversations about PICS.

Prior studies have provided insight on PICS awareness and communication, but few to our knowledge have included both providers and patients in a general ICU cohort. Detsky and colleagues^24^ found that 39% of physicians (117/300) and 31% of nurses (92/300) reported having conversations regarding long-term impairments with patients although almost all (84% and 90%, respectively) had thought about future morbidity. White and colleagues^23^ reported that 86% of 51 recorded provider-family meetings included discussion of future functional status or quality of life. However, this study focused on meetings for end-of-life conversations, withdrawal of treatment, or delivering bad news,^23^ which does not provide data on conversations for patients expected to recover and for whom these conversations might be most helpful. Govindan and colleagues^25^ reported survey results collected at a conference of Michigan hospitals showing that, similar to the results reported here, only about one-third of providers (34%) routinely communicate with patients or families about survivorship.^25^

Improving communication about PICS is pivotal for the well-being of ICU survivors for several reasons. As previously described by Turnbull and colleagues,^34^ expectations for recovery after critical illness have a key impact on quality of life. Quality of life has long been described in terms of a person’s perceived health in relationship to their expectations.^35^, ^36^, ^37^ When patients experience lower objective health (eg, impairments), but psychologically are able to adapt to and accept this health as a new baseline, they may experience a response shift.^37^^,^^38^ Recalibration and reprioritization are 2 aspects of a response shift.^34^ Recalibration is the ability to change one’s internal standards while reprioritization is one’s ability to change personal values. For example, consider an older woman who previously played bridge with friends and was always fiercely competitive. She recently survived septic shock and feels cognitively slower than before. Recalibration would be accepting that her brain might not process as quickly now, and a third-place finish is worthy of satisfaction. Reprioritization would be focusing on the joys of being with friends regardless of how she plays. Both have been hypothesized to increase quality of life as survivors assimilate to a new baseline. In other populations, this has been demonstrated in the disability paradox,^39^ wherein people with chronic disabilities report being more happy, on average, than expected.^40^ In older adults, those who have a realistic acceptance of future health decline have less depression than those who have an unrealistic optimism.^41^ Patients who have undergone knee surgery who falsely believe that their recovery will be quick are less satisfied than those who accept from the onset that recovery might be slow.^42^

However, paramount in manifesting a response shift and managing expectations for ICU survivors is having an awareness and understanding of the likely trajectory of one’s health after critical illness. An ICU survivor who expects to return to their baseline before the illness is likely to have difficulty assimilating to months or years with PICS if they do not know PICS exists. This is consistent with qualitative studies of patients who survive an ICU stay. Thematic analyses of patient interviews have shown that normalization and expectation management are considered by patients and caregivers to be important elements of follow-up care and positive transition experiences.^43^, ^44^, ^45^ Similarly, in a cohort of adult survivors of acute respiratory failure, those who met their expectations for recovery reported a significantly higher quality of life than those who did not meet their expectations.^46^ Studies have demonstrated that roughly one-half of patients who survive an ICU stay experience a discrepancy between their perceived health and their predicted health,^47^^,^^48^ further emphasizing the role that anticipatory guidance could play in managing expectations to improve future quality of life.

Strengths of this study relate to the sample size and ability to collect patient characteristics. We were able to obtain > 500 total surveys from a wide variety of patients, provider roles, and ICU types. The institutions through which we distributed the surveys are geographically diverse and represent both academic and community hospitals. The patient group showed a similar burden of PICS to other general ICU cohorts of respiratory failure or shock survivors in that roughly 1 in 3 patients reported struggling with each of the 3 domains of PICS and about one-half of patients struggled with at least 1 domain of PICS.^18^ Our results are similar to other studies of survivorship communication,^24^^,^^25^ but add to the literature in its larger size^25^ and inclusion of patient experiences.^24^

Several limitations should be noted. We were unable to calculate a response rate for providers; however, we assume this rate was low overall. Providers largely were from medical ICUs, whereas patients were admitted mostly to surgical or cardiovascular ICUs. Because of email distribution availability, we did not target other important multidisciplinary providers (pharmacists; respiratory, physical, or occupational therapists; speech language pathologists; and others) who might engage in these PICS conversations and who should be part of these conversations. We did not record time from discharge until survey completion; although recall bias could affect patient responses, this further emphasizes the need for communication interventions that improve delayed recall. Respondents were almost all White, and because of our survey design, we included only patients with English as their documented written language. Providers were self-selected and patients were invited from an institution known to perform extensive PICS research, but both selection biases likely would inflate responses toward more communication, awareness, and recall. Finally, our survey was created with feedback from qualitative research experts and ICU clinicians, but has not been validated.

Our study highlights the PICS communication gap (Fig 2), including several targets for provider education and training (eg, targeting RN awareness and tools for confidence of all providers). We are limited on aspects of the patient-caregiver side of our proposed communication pathway (Fig 1), which warrants further investigation, in particular understanding how families and caregivers receive, understand, and remember this information. Future research could include assessment of immediate recall and comprehension when communication about survivorship is known to have occurred, compared with delayed recall and comprehension. No consensus was found among provider respondents as to who currently has the responsibility of discussing recovery trajectories and possible health problems with patients and families. We suggest that the choice of responsible provider(s), ideal timing, and optimal strategies for improved communication remain unanswered questions,^49^ although approaches have been proposed for how to proceed pending further studies.^50^

## Interpretation

This survey-based study demonstrated that awareness of PICS is inconsistent among providers and poor among nurses and patients. Providers did not have strong confidence in communicating PICS, and less than one-third of them reported doing so at least one-half of the time. About 1 in 3 patients remembered being told that they might struggle with PICS and its various domains, although almost one-half reported symptoms suggestive of PICS. This warrants further investigation to determine by whom, when, and how these conversations should occur.

## Funding/Support

This project was supported by the 10.13039/100006108National Center for Advancing Translational Sciences [Clinial and Translational Science Award UL1 TR002243]. M. L. R. is supported by the 10.13039/100000050National Heart, Lung, and Blood Institute [Grant T32HL087738]. E. W. E. is supported by the 10.13039/100000049National Institute on Aging [Grant R01AG058969]. E. W. E. and M. F. M. are supported by the US Department of Veteran Affairs [Grants I01RX002992 and IK2RX004799, respectively).

## Financial/Nonfinancial Disclosures

None declared.

## Supplementary Data

e-Online Data
