# Supplementary material for: Post-Intensive Care Syndrome Awareness and Communication: Surveys of ICU Providers and Patients
Source: Chest. 2025 Oct 10;169(2):486–95. doi: 10.1016/j.chest.2025.08.048 (PMC12895331; doi:10.1016/j.chest.2025.08.048)
Supplement: e-Online Data [file mmc1.docx]

**Supplement**

1. **Full provider survey**
2. **Full patient survey**
3. **Additional results of provider surveys**
4. **Additional results of patient surveys**

1. Provider surveys

------------------------------------------------------------------------------------------------------------------

Survey for Post Intensive Care Syndrome

Thank you for agreeing to participate in this research survey, please do not hesitate to reach out with questions. ***

This is voluntary, there is no direct benefit to you and no alternative to participating in the study. There is no compensation. Your answers will not be linked to your identity and will not affect your employment. You may stop at any time during the survey and there is no penalty for not completing.

By filling out this survey, you are agreeing to let us use your answers for research purposes. You may skip questions or stop at any time.

1. What is your role in the ICU?

O RN

0 Attending

O APP

O Fellow

1. What ICU do you primarily work in?

0 Burn ICU

0 CV ICU

0 Medical ICU

0 Neuro ICU

0 Surgical ICU

0 Trauma ICU

O Combination of 2 or more

O other

1. Before this survey, have you heard the term Post Intensive Care Syndrome?

0 Yes

0 No

1. It is my understanding that of ICU survivors will have new or worsening impairments in cognitive, physical, or mental health function > 3 months after ICU discharge

0 < 20%

0 21-50%

0 51-80%

0 81-100%

For the following questions, please imagine you are taking care of a patient who was admitted to your unit. They were on mechanical ventilation and vasopressors, and experienced delirium for several days. They are now recovering well and not delirious, off all support and about to leave the ICU. You suspect they will return home after the hospital and maybe even return to employment.

1. How often would you counsel this patient or their family about new or worsening impairments that might occur following critical illness?

O All or almost all the time

O Most of the time (more than half)

O Some of the time (less than half)

O Seldom or Never

1. How often would you tell this patient or their family about the potential for cognitive impairment following critical illness (e.g. difficulty with concentration or memory)?

O All or almost all the time

O Most of the time (more than half)

O Some of the time (less than half)

O Seldom or Never

1. How often would you tell this patient or their family about the potential for physical impairment following critical illness (e.g. decreased mobility)?

O All or almost all the time

O Most of the time (more than half)

O Some of the time (less than half)

O Seldom or Never

1. How often would you tell this patient or their family about the potential for mental health impairment following critical illness (e.g. depression, anxiety or PTSD)?

O All or almost all the time

O Most of the time (more than half)

O Some of the time (less than half)

O Seldom or Never

1. What is your confidence level in answering questions regarding the above-mentioned impairments when asked by patients or their families about the likelihood of these occurring?

O Poor

O Somewhat good

O Good

0 Excellent

1. My ICU considers it important to talk with patients and their families about impairments and challenges that can occur during recovery in the months to years following critical illness

O Strongly Disagree

O Disagree

O Agree

O Strongly Agree

1. Who in your ICU, if anyone, currently has responsibility for having discussions with patients and caregivers regarding impairments in physical, cognitive, and mental health that might occur after the ICU?
2. RNs

O House staff (including residents and fellows)

1. APPs

O Attendings

O Physical Therapy or Occupational Therapy

O Palliative Care

1. Hospitalists

O No one currently has responsibility

O Other

1. Patient Surveys

-----------------------------------------------------------------------------------------------------------------

Patient Experiences of ICU Communication

1. In which ICU were you most recently admitted?

0 Burn ICU

O Cardiovascular ICU

0 Medical ICU

0 Neuro ICU

0 Surgical ICU

0 Trauma ICU

O I am not sure

O Other

1. Do you remember ever being told the term Post Intensive Care Syndrome during any hospitalization in the past year?

O Yes

0 No

1. I am currently living in or located at:

O My house or apartment

O The house or apartment of a family member or friend

O A skilled nursing facility (SNF)

O A long-term acute care facility (LTAC)

O Another type of rehabilitation facility

O none of the above

1. Is this different than where you lived before your last hospitalization?

O Yes

0 No

The following questions refer to any point during the most recent hospital admission that included being in the Intensive Care Unit (ICU)

1. I remember being told that I might not return to the same baseline function (including thinking, physical abilities or mental health) after the ICU admission as I was before the ICU.

O Yes

0 No

1. I remember being told that I might develop new or worsening difficulty with concentration, memory, or mental tasks in the months to years following critical illness.

O Yes

1. No
2. I remember being told that I might develop new or worsening difficulties with walking, strength or physically performing daily tasks in the months to years following critical illness.

O Yes

1. No
2. I remember being told that I might develop new or worsening mental health such as anxiety, depression, or PTSD in the months to years following critical illness.

O Yes

1. No
2. I am surprised at how challenging the recovery from critical illness has been.

O Yes

1. No
2. Now I am close to functioning at the same level as before the ICU admission.

O Yes

1. No
2. I have struggled with new or worsening concentration, memory or mental tasks following the ICU.

O Yes

1. No
2. I have struggled with new or worsening walking, strength or physically performing daily tasks.

O Yes

1. No
2. I have struggled with new or worsening mental health such as anxiety or depression following the ICU.

O Yes

0 No

1. I wish there were more conversations about recovery from critical illness before leaving the ICU.

0 Strongly Agree

0 Agree

0 Disagree

0 Strongly Disagree

1. What specifically, if anything, do you wish you would have been told before leaving the hospital about the recovery from critical illness, the possible impairments, and challenges?
2. Do you wish to receive a $20 Amazon gift card by email? These will typically be sent within 2 weeks to the same email used for this survey link.
3. Additional Provider Survey Results

----------------------------------------------------------------------------------------------------------------

Responses per institution:

Question 4: It is my understanding that of ICU survivors will have new or worsening impairments in cognitive, physical, or mental health function > 3 months after ICU discharge

1. Question 11: Who in your ICU, if anyone, currently has responsibility for having discussions with patients and caregivers regarding impairments in physical, cognitive, and mental health that might occur after the ICU?

*Our study describes responses from providers at 9 institutions and patients at 1 institution (Vanderbilt University Medical Center, Nashville TN [VUMC]). Below, we provide comparison of providers at VUMC (i.e. same institution as patient respondents; N=150) vs other institutions (N=232)*

**Question 3:**

“Before this survey, have you heard the term Post Intensive Care Syndrome?”

- VUMC: 71.4% ‘Yes’ (105/147)
- Non-VUMC: 75.3% ‘Yes’ (171/227)

**Question 5:**

1. Additional Patient Survey Results

-----------------------------------------------------------------------------------------------------------------

Question #3:

Question #4:

*Comparison of patients with adjudicated acute respiratory failure or shock and those without.*

**Question 2:** “Do you remember ever being told the term Post Intensive Care Syndrome during any hospitalization in the past year?”

Acute Respiratory Failure or Shock: 16.9% (20 of 118 responses)

ICU admission without Acute Respiratory Failure or Shock: 14.8% (4 of 27 responses)

**Question 5**: “I remember being told that I might not return to the same baseline function (including thinking, physical abilities or mental health) after the ICU admission as I was before the ICU.”

Acute Respiratory Failure or Shock: 35.3% (42 of 119 responses)

ICU admission without Acute Respiratory Failure or Shock: 37% (10 of 27 responses)

**Question 14:** “I wish there were more conversations about recovery from critical illness before leaving the ICU.”

Acute Respiratory Failure or Shock: 58.0% “Agree or Strongly Agree” (69 of 119 responses)

ICU admission without Acute Respiratory Failure or Shock: 59.3% (16 of 27 responses)
